# Overexpression of *Tetrahymena* Cysteine Synthetase 1 Promotes Cadmium Removal by Biosynthesizing Cadmium Sulfide Quantum Dots in *Escherichia coli*

**DOI:** 10.3390/ijms26083685

**Published:** 2025-04-13

**Authors:** Wenliang Lei, Juan Liu, Yiwei Liu, Jing Xu, Wei Wang

**Affiliations:** 1 Key Laboratory of Chemical Biology and Molecular Engineering of Ministry of Education, Institute of Biotechnology, Shanxi University, Taiyuan 030006, China; 202113002002@email.sxu.edu.cn (W.L.); 202213002004@email.sxu.edu.cn (J.L.); 202323002014@email.sxu.edu.cn (Y.L.); 2 School of Life Science, Shanxi University, Taiyuan 030006, China; 3 Shanxi Key Laboratory of Biotechnology, Taiyuan 030006, China

**Keywords:** cadmium, cysteine synthetase 1, biomineralization, quantum dots, protein overexpression, *Escherichia coli*

## Abstract

Heavy metal cadmium causes significant contamination in aquatic ecosystems. The biomineralization of cadmium represents a vital biological mechanism for handling cadmium stress in diverse microorganisms. To improve the biomineralization capacity of cadmium by microorganisms in aquatic environments, *Tetrahymena* cysteine synthetase 1 (TtCsa1) was overexpressed in *E. coli*. The tolerance of *E. coli*/pET-28a-*TtCSA1* to cadmium was enhanced by expressing TtCsa1. Upon addition of cysteine, *E. coli*/pET-28a-*TtCSA1* generated more H_2_S, which reacted with Cd^2+^ to form CdS quantum dots (QDs), resulting in a stronger fluorescence signal. The UV-visible absorption and fluorescence spectra of the culture supernatant of *E. coli*/pET-28a-*TtCSA1* showed characteristic peaks corresponding to CdS QDs. Transmission Electron Microscopy (TEM) images confirmed that the formation of CdS QDs and their agglomeration in the *E. coli* cells. X-ray Diffraction Analysis (XRD) analysis further confirmed the presence of QDs and their crystalline nature. In rich medium, *E. coli*/pET-28a-*TtCSA1* achieved removal rates of 99.5%, 98.2%, 56.5%, and 49.4%, respectively, for Cd^2+^ concentrations of 0.15, 0.3, 0.45, and 0.6 mM within 48 h. In simulated wastewater, *E. coli*/pET-28a-*TtCSA1* achieved removal rates of 99.4%, 94.3%, 90.1%, and 89.8%, respectively, for Cd^2+^ concentrations of 0.3, 0.45, 0.6, and 0.75 mM within 12 h. These results demonstrate that overexpressing TtCsa1 in *E. coli* can significantly enhance its ability to biomineralize Cd^2+^ in rich medium and simulated wastewater, which has potential applications in bioremediation of aquatic environments contaminated with heavy metals.

## 1. Introduction

With the rapid development of industry and the expansion of commercial applications, environmental heavy metal pollution has become increasingly severe [1,2,3]. Among these pollutants, cadmium is a highly toxic heavy metal that has caused significant contamination in aquatic ecosystems. It disrupts the normal physiological activities of organisms, accumulates through the food chain, and poses serious threats to human health, including a potential contribution to cancer [4,5,6]. Due to the non-biodegradable nature of cadmium, its removal from water bodies is challenging once it enters these ecosystems, underscoring the urgency of developing effective removal methods. Consequently, the development of efficient strategies for mitigating cadmium pollution in the environment has garnered widespread global attention [7,8,9].

Various methods have been employed to remove cadmium from aquatic environments, including chemical precipitation, physical adsorption, and biosorption [7,10,11]. These traditional methods are effective, but often lead to high resource consumption and potential for secondary environmental pollution. In contrast, bioremediation, modulating the metabolic pathways of microorganisms, has gained significant attention due to its cost-effectiveness and environmental friendliness [12]. Consequently, extensive research has been conducted to explore more efficient biological approaches for cadmium removal. The biological removal of cadmium primarily relies on metabolic pathways inherent to microorganisms, such as biosorption, biotransformation, and biomineralization [13,14]. Notably, biomineralization produces cadmium sulfide quantum dots (CdS QDs) while removing cadmium from the environment [15,16].

Various strategies have been developed to enhance microbial biomineralization, among which cysteine is a commonly used exogenous additive [17,18]. This amino acid serves as a crucial precursor for the synthesis of hydrogen sulfide (H_2_S) [19,20]. The resulting H_2_S plays a pivotal role in mitigating heavy metal stress by forming metal sulfides in various microorganisms [21,22,23]. Under cadmium stress, deep-sea bacterium *Idiomarina* sp. OT37-5b produces CdS QDs on its cell surfaces. The addition of L-cysteine not only enhances cell survival but also achieves nearly 99% cadmium removal [24,25]. Similarly, the direct addition of sodium sulfide, an exogenous H_2_S donor, promotes the generation of more CdS QDs in *Tetrahymena thermophila* [7]. When *Rhodopseudomonas palustris* is co-stressed with cadmium and cysteine, the cells primarily depend on biomineralization to generate CdS QDs, facilitating the removal of cadmium from the aquatic environment [26]. These findings show that cysteine not only protects microorganisms from heavy metal stress but also promotes the efficient production of CdS QDs.

The metabolic pathways inherent to microorganisms are frequently limited by intracellular enzyme levels and microbial activity. To enhance the substrate utilization capacity of microorganisms, the overexpression of proteins through engineered microbes represents a viable strategy [27,28,29]. For instance, the overexpression of glutathione synthetase in yeast has been demonstrated to significantly enhance the biomineralization capacity of cadmium [30]. Similarly, transgenic tobacco that overexpressed cysteine synthase exhibited increased tolerance to heavy metals [31]. *Escherichia coli* exhibits strong environmental adaptability and is widely utilized for protein overexpression due to its modifications for enhanced performance [32]. The overexpression of glutathione synthetase enzymes GshA and GshB in *E. coli* simultaneously increases cadmium tolerance and bioaccumulation. The overexpression of cysteine desulfurase significantly increases the production of CdS QDs, which can be applied in photocatalytic dye degradation. Therefore, the construction of suitable engineered strains would be highly beneficial for advancing practical applications in cadmium removal and related fields [33].

In our previous studies, we identified a *Tetrahymena* cysteine synthase (TtCsa1) that utilizes cysteine as a substrate for the synthesis of H_2_S. *TtCSA1* knockdown also decreases the intracellular synthesis of cysteine and glutathione, which consequently impairs the ability of cells to resist heavy metal stress [34]. H_2_S synthesis plays a critical role in enabling *Tetrahymena* to cope with cadmium stress by promoting the formation of CdS QDs [35]. TtCsa1 also drives the biomineralization of CdS QDs in vitro [36]. However, it has not been confirmed whether the overexpression of TtCsa1 can enhance the ability of cells to mineralize and produce CdS. *Tetrahymena* is not a well-established system for protein engineering and large-scale applications. To address this limitation, TtCsa1 was overexpressed in *E. coli*. The engineered *E. coli*/pET-28a-*TtCSA1* significantly enhanced the cadmium tolerance. Upon the addition of cysteine, *E. coli*/pET-28a-*TtCSA1* generated more H_2_S, which reacted with Cd^2+^ to form CdS QDs. It effectively removed cadmium contamination in LB medium and simulated wastewater. The findings offer a promising way for developing more efficient cadmium-resistant microorganisms for environmental cleanup and for understanding the biological mechanisms of heavy metal tolerance in microorganisms.

## 2. Results

### 2.1. Overexpression of TtCsa1 Significantly Enhances Cadmium Resistance in E. coli

**Figure 1:**
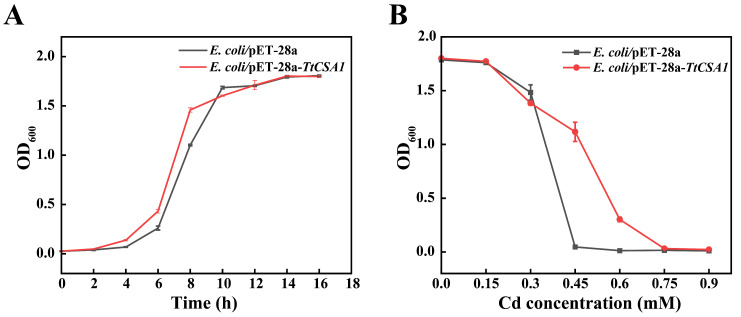
Overexpression of His-Csa1 improves Cadmium Tolerance in *E. coli*. (**A**) Proliferation of *E. coli/*pET-28a and *E. coli*/pET-28a-*TtCSA1.* (**B**) Minimum inhibitory concentration (MIC) assay of cadmium ions for *E. coli/*pET-28a and *E. coli*/pET-28a-*TtCSA1*.

TtCsa1 exhibits enzymatic activity for H_2_S production in *T*. *thermophila* [34]. The expression of *TtCSA1* is significantly upregulated in the presence of cadmium. TtCsa1 plays a crucial role in biomineralization-based detoxification of cadmium in *T. thermophila* [33]. To investigate whether the heterologous expression of TtCsa1 enhances *E. coli* ’s biomineralization capability, TtCsa1 was expressed in *E. coli*/pET-28a-*TtCSA1*. SDS-PAGE analysis showed the soluble expression of His-Csa1 in *E. coli*/pET-28a-*TtCSA1* (Figure S1). There was no significant difference in the viability of *E. coli*/pET-28a and *E. coli*/pET-28a-*TtCSA1* after IPTG induction (Figure 1A). After induction with IPTG, *E. coli*/pET-28a and *E. coli*/pET-28a-*TtCSA1* were subjected to 24 h of cadmium ion stress at varying concentrations. The results indicated that the minimum inhibitory concentration (MIC) of *E. coli*/pET-28a was 0.6 mM, while the MIC for *E. coli*/pET-28a-*TtCSA1* was 0.9 mM (Figure 1B).

### 2.2. TtCsa1 and Cysteine Enhance Biomineralization of Cd in E. coli

**Figure 2:**
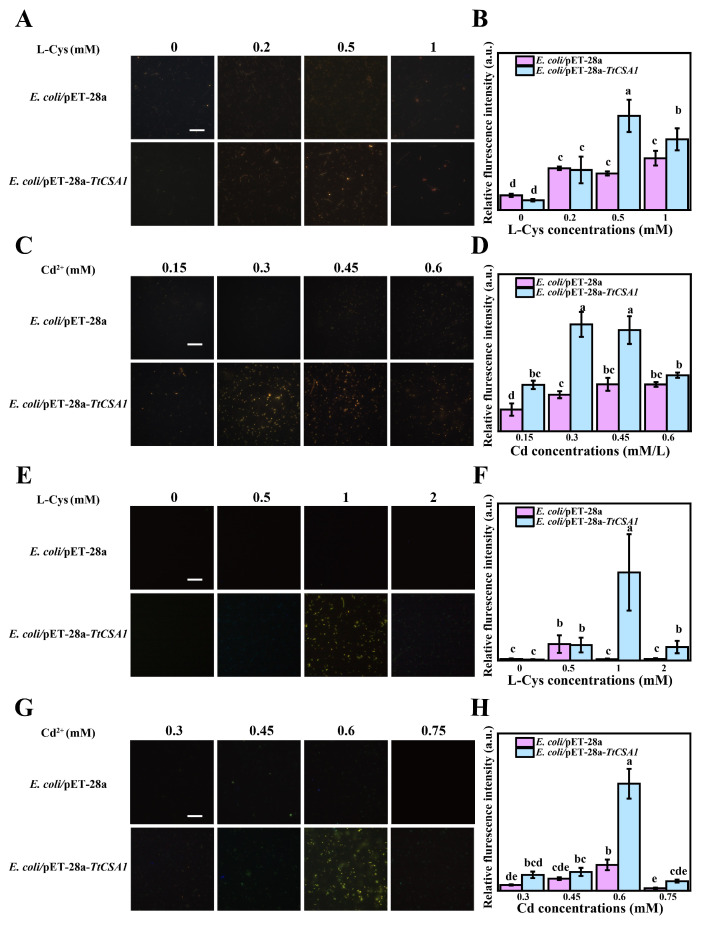
Overexpression of His-*TtCSA1* in *E. coli* catalyzes the formation of quantum dots in rich medium and wastewater. (**A**) The formation of quantum dots was observed after incubating *E. coli*/pET-28a and *E. coli*/pET-28a-*TtCSA1* in LB medium containing 0.45 mM cadmium and varying concentrations of L-cysteine (0, 0.2, 0.5, and 1 mM) for 48 h. Scale bar, 20 µm. (**B**) Relative fluorescence intensity analysis of QDs in *E. coli* after 48 h of incubation, corresponding to the conditions shown in (**A**) (*n* = 300). (**C**) The formation of quantum dots was observed after incubating *E. coli*/pET-28a and *E. coli*/pET-28a-*TtCSA1* with 0.5 mM L-cysteine and varying concentrations of cadmium (0, 0.15, 0.3, 0.45, and 0.6 mM) in LB medium for 48 h. Scale bar, 20 µm. (**D**) Relative fluorescence intensity analysis of QDs in E. coli after 48 h of incubation, corresponding to the conditions showed in (**C**) (*n* = 300) (**E**) The formation of quantum dots was observed after incubating *E. coli*/pET-28a and *E. coli*/pET-28a-*TtCSA1* in wastewater containing 0.3 mM cadmium and varying concentrations of L-cysteine (0, 0.5, 1, and 2 mM) for 12 h. Scale bar, 20 µm. (**F**) Relative fluorescence intensity analysis of QDs in E. coli after 12 h of incubation, corresponding to the conditions shown in (**E**) (*n* = 300) (**G**). The formation of quantum dots was observed after incubating *E. coli*/pET-28a and *E. coli*/pET-28a-*TtCSA1* with 1 mM L-cysteine and varying concentrations of cadmium (0.3, 0.45, 0.6, 0.75, and 0.9 mM) for 12 h in wastewater. Scale bar, 20 µm. (**H**) Relative fluorescence intensity analysis of QDs in *E. coli* after 12 h of incubation, corresponding to the conditions shown in (**G**) (*n* = 300). Data are means ± SE of three biological repeats, error bars indicate error standard. Means denoted by the same letter were not significantly different at *P* > 0.05, and different letters indicate statistically significantly differences (*p* < 0.05) by Duncan Multiple Range Test (DMRT).

**Figure 3:**
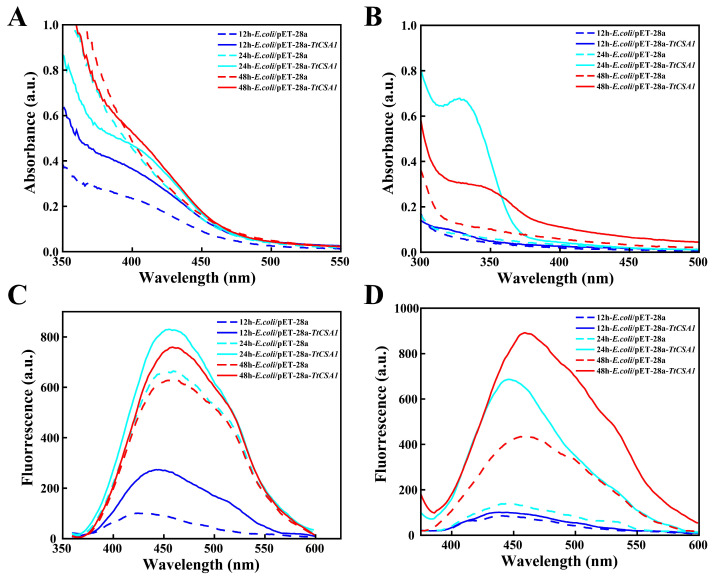
Spectral analysis of CdS generated using *E. coli*/pET-28a and *E. coli*/pET-28a-*TtCSA1*. (**A**) UV-Visible absorption spectra of the supernatants obtained after centrifugation of *E. coli*/pET-28a and *E. coli*/pET-28a-*TtCSA1*, incubated in LB medium containing 0.45 mM Cd^2+^ and 0.5 mM L-cysteine for 12, 24, and 48 h. (**B**) Corresponding fluorescence spectra for the samples described in (**A**). (**C**) UV-Visible absorption spectra of the supernatants obtained after centrifugation of *E. coli*/pET-28a and *E. coli*/pET-28a-*TtCSA1* incubated in wastewater containing 0.6 mM Cd^2+^ and 1 mM L-cysteine for 12, 24, and 48 h. (**D**) Corresponding fluorescence spectra for the samples described in (**C**).

Previous studies have demonstrated that cysteine enhances the biomineralization capacity of *Tetrahymena* cells [7]. Under 0.45 mM cadmium stress, *E. coli*/pET-28a and *E. coli*/pET-28a-*TtCSA1* were exposed to different concentrations of L-cysteine. The fluorescence intensity of QDs produced by *E. coli*/pET-28a-*TtCSA1* was stronger compared to that in *E. coli*/pET-28a. The highest QD production was observed in *E. coli*/pET-28a-*TtCSA1* incubating with 0.5 mM L-cysteine (Figure 2A,B). Furthermore, *E. coli*/pET-28a-*TtCSA1* formed more QDs compared to *E. coli*/pET-28a at different concentrations of Cd^2+^ (Figure 2C,D). Bright-field imaging also revealed that the biomineralization particles were distributed at the cellular poles and extensively across other regions of the cells in *E. coli*/pET-28a-*TtCSA1* (Figure S2A). The UV-visible absorption and fluorescence spectra of the culture supernatant of *E. coli*/pET-28a-*TtCSA1* exhibited a redshift with prolonged incubation time, indicating an increase in the size of CdS QDs. The intensity of the UV absorption and fluorescence emission peaks in *E. coli*/pET-28a-*TtCSA1* was higher than that in *E. coli*/pET-28a, suggesting that TtCsa1 promotes CdS QD production (Figure 3A,B).

To evaluate the biomineralization capability of *E. coli*/pET-28a-*TtCSA1* in wastewater, environmental water samples from the Fen River in Taiyuan were used to simulate heavy metal-contaminated wastewater. When the cells were treated with 0.3 mM cadmium ions and different concentrations of L-cysteine, the fluorescence intensity of CdS QDs produced by *E. coli*/pET-28a-*TtCSA1* was significantly higher than that of *E. coli*/pET-28a (Figure 2E,F). Bright-field imaging revealed that *E. coli*/pET-28a-*TtCSA1* produced significantly more particles compared to *E. coli*/pET-28a (Figure S2B). The fluorescence intensity of CdS QDs generated by *E. coli*/pET-28a-*TtCSA1* was consistently higher than that in *E. coli*/pET-28a (Figure 2G,H). Furthermore, UV-visible absorption and fluorescence spectra of the culture supernatant indicated the presence of CdS peaks in the supernatant of *E. coli*/pET-28a-*TtCSA1* (Figure 3C,D).

### 2.3. E. coli/pET-28a-TtCSA1 Produces More H_2_S upon the Addition of Cysteine

**Figure 4:**
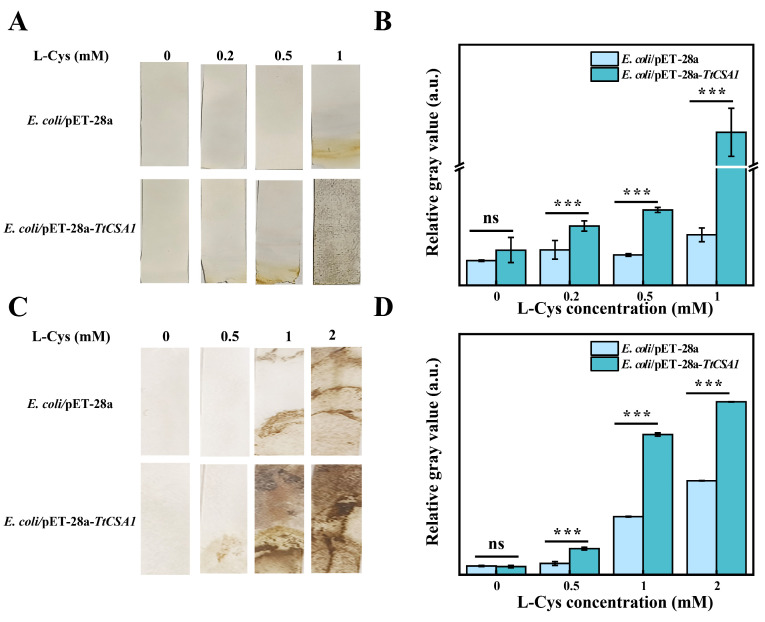
Production of hydrogen sulfide in *E. coli* /pET-28a and *E. coli*/pET-28a-*TtCSA1*. (**A**) Detection of H_2_S generation using lead acetate paper after incubating *E. coli*/pET-28a and *E. coli*/pET-28a-*TtCSA1* in rich medium containing 0.45 mM cadmium ions and varying concentrations of L-cysteine (0, 0.2, 0.5, and 1 mM) for 12 h. (**B**) Relative gray intensity analysis in (**A**). (**C**) Detection of H_2_S generation using lead acetate paper after incubating *E. coli*/pET-28a and *E. coli*/pET-28a-*TtCSA1* with 0.3 mM cadmium ions and varying concentrations of L-cysteine (0, 5, 1, and 2 mM) in wastewater for 24 h. (**D**) Relative gray intensity analysis in (**C**). The symbols ns and *** represent non-significance (*p* > 0.05) and extremely significant differences (*p* < 0.01), respectively.

Cadmium can enter cells through mechanisms such as ingestion and ion channels, causing harm to the cells [37,38]. The biomineralization of CdS through H_2_S is an important mechanism for reducing cadmium toxicity [16]. We further investigated the production of H_2_S in *E. coli*. In the LB rich medium, *E. coli*/pET-28a-*TtCSA1* produced more H_2_S than *E. coli*/pET-28a upon the addition of L-cysteine (Figure 4A,B). In simulated wastewater, *E. coli*/pET-28a-*TtCSA1* also produced more H_2_S than *E. coli*/pET-28a upon the addition of L-cysteine (Figure 4C,D). When different concentrations of cadmium were added to LB containing 0.5 mM L-cysteine, a decrease in detected H_2_S was observed with increasing cadmium concentration (Figure S3A,B). A similar decrease in detected H_2_S with increasing cadmium concentration was also observed in simulated wastewater (Figure S3C,D). These results imply that more CdS QDs were formed in the *E. coli/*pET-28a-*TtCSA1* with increasing cadmium concentrations.

### 2.4. Characterization of CdS Quantum Dots in E. coli

**Figure 5:**
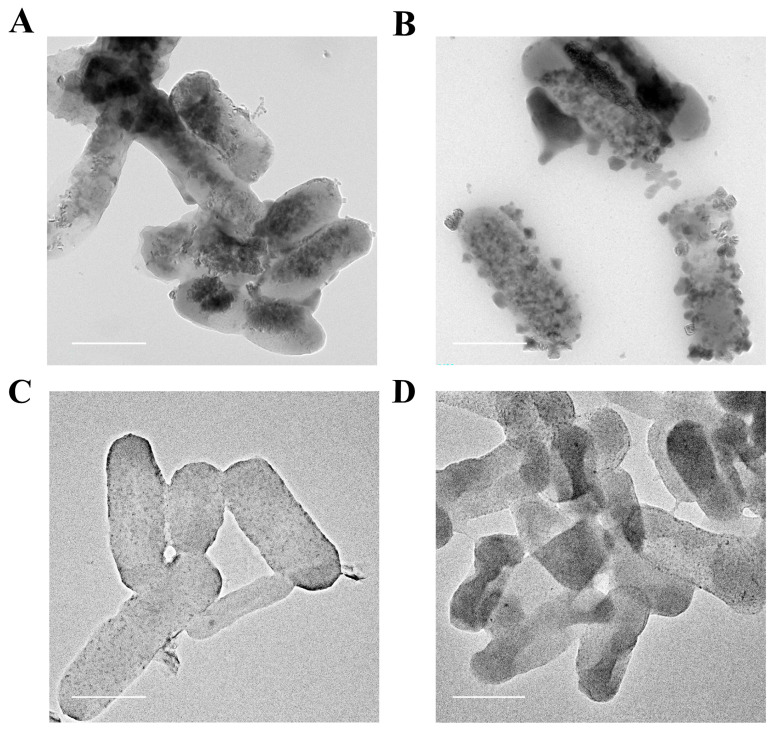
Transmission Electron Microscopy (TEM) observation of *E. coli* treated with cadmium and cysteine. (**A**) The formation of cadmium sulfide was shown using TEM in *E. coli*/pET-28a after 48 h of incubation in LB medium. Scale bar, 1 μm. (**B**) The formation of cadmium sulfide was shown by TEM in *E. coli*/pET-28a-*TtCSA1* after 48 h of incubation in LB medium. Scale bar, 1 μm. (**C**) The formation of cadmium sulfide was observed by TEM in *E. coli*/pET-28a after 48 h of incubation in wastewater. Scale bar, 1 μm. (**D**) The formation of cadmium sulfide was observed by TEM in *E. coli*/pET-28a-*TtCSA1* after 48 h of incubation in wastewater. Scale bar, 1 μm.

**Figure 6:**
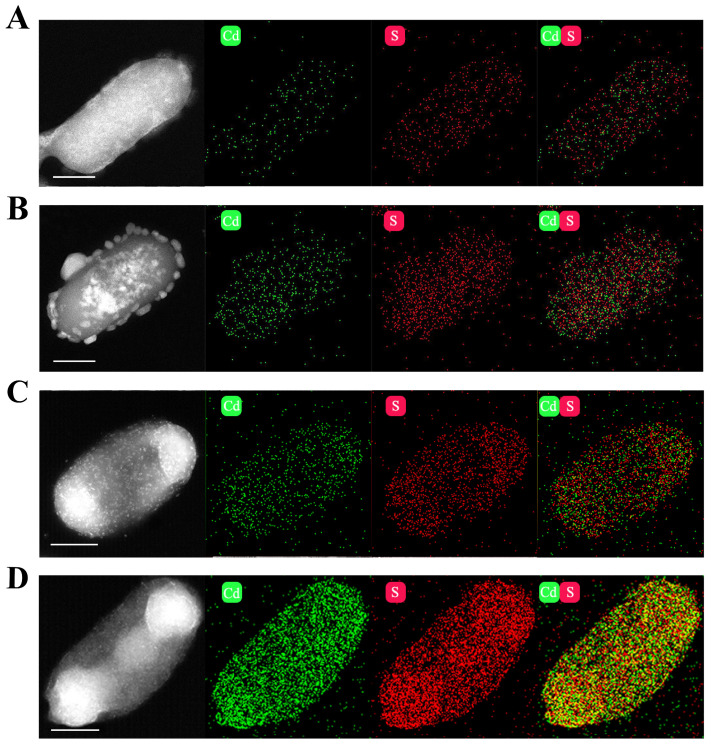
Energy dispersive X-ray (EDX) spectroscopy analysis of cadmium sulfide quantum dot generated in *E. coli*. (**A**) EDX spectroscopy analysis showing the elemental distribution on the surface of *E. coli*/pET-28a after incubation for 48 h in LB medium containing 0.45 mM cadmium ions and 0.5 mM L-cysteine. Scale bar, 500 nm. (**B**) EDX spectroscopy analysis showing the elemental distribution on the surface of *E. coli*/pET-28a-*TtCSA1* after incubation for 48 h in LB medium containing 0.45 mM cadmium ions and 0.5 mM L-cysteine. Scale bar, 500 nm. (**C**) The elemental distribution analysis was shown using EDX spectroscopy in *E. coli*/pET-28a after 48 h of incubation in wastewater containing 0.6 mM cadmium ions and 1 mM L-cysteine. Scale bar, 500 nm. (**D**) The elemental distribution analysis was shown using EDX spectroscopy in *E. coli*/pET-28a-*TtCSA1* after 48 h of incubation in wastewater containing 0.6 mM cadmium ions and 1 mM L-cysteine. Scale bar, 500 nm.

*E. coli*/pET-28a and *E. coli*/pET-28a-*TtCSA1* maintained normal cell morphology following 48 h of cadmium ion stress and cysteine incubation in both rich medium and simulated wastewater (Figure S4). High-resolution Transmission Electron Microscopy (TEM) demonstrated that *E. coli*/pET-28a-*TtCSA1* exhibited greater attachment of CdS particles compared to *E. coli*/pET-28a in rich medium (Figure 5A,B). Furthermore, larger aggregates of cadmium sulfide particles were attached to the surface of *E. coli*/pET-28a-*TtCSA1* in wastewater (Figure 5C,D). Energy Dispersive Spectroscopy (EDS) mapping also indicated that *E. coli*/pET-28a-*TtCSA1* contained higher amounts of cadmium and sulfur elements than *E. coli*/pET-28a (Figure 6A–D). Interestingly, *E. coli*/pET-28a-*TtCSA1* contained higher amounts of cadmium and sulfur elements in wastewater than in rich medium (Figure 6B,D). X-ray diffraction analysis (XRD) indicated that the cadmium sulfide produced by *E. coli*/pET-28a in LB and simulated wastewater did not match the standard CdS pattern, whereas the XRD pattern of the cadmium sulfide produced by *E. coli*/pET-28a-*TtCSA1* was attributed to the sphalerite type. The diffraction peaks at 28.217° and 32.778° corresponded to the (111) and (220) crystal planes of CdS (JCPDS No. 21-0829) (Figure S5) [39].

### 2.5. Removal of Cadmium Ions from Rich Medium

**Figure 7:**
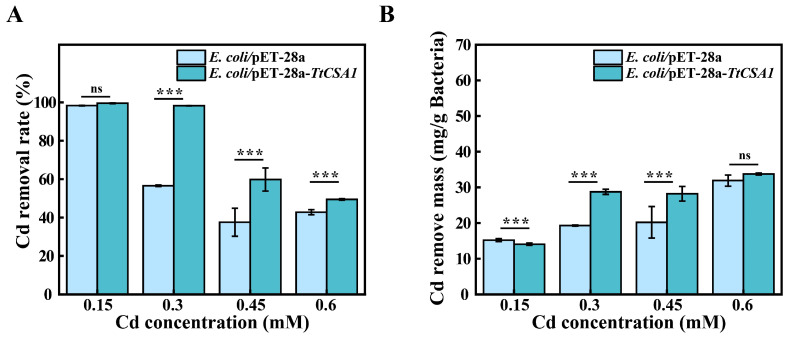
*E. coli*/pET-28a-*TtCSA1* effectively remove Cadmium in rich medium. (**A**) Removal rates of cadmium at different concentrations by *E. coli*/pET-28a and *E. coli*/pET-28a-*TtCSA1* in LB medium supplemented with 0.5 mM L-cysteine. (**B**) Mass of cadmium removed per gram of dry weight of the biomass from the experiments in (**A**). The symbols ns and *** represent non-significance (*p* > 0.05) and extremely significant differences (*p* < 0.01), respectively.

*E. coli*/pET-28a-*TtCSA1* formed CdS QDs in a rich medium. To evaluate the removal rates of Cd^2+^, the cadmium content in the medium was determined using ICP-OES/MS. *E. coli*/pET-28a-*TtCSA1* and *E. coli*/pET-28a achieved removal rates of 99.5% and 98.3%, respectively, for 0.15 mM Cd^2+^ (Figure 7A). *E. coli*/pET-28a-*TtCSA1* achieved 98.2% and 59.8% removal rates for 0.3 mM and 0.45 mM cadmium solutions, respectively, with the removal mass per gram of dry weight bacteria being 28.7 mg and 28.2 mg, respectively. *E. coli*/pET-28a only achieved removal rates of 56.5% and 37.5% for 0.3 mM and 0.45 mM cadmium solutions, respectively, with the removal mass per gram of dry weight bacteria being 19.3 mg and 20.2 mg, respectively (Figure 7B). For 0.6 mM Cd^2+^ solutions, *E. coli*/pET-28a-*TtCSA1* and *E. coli*/pET-28a removed 49.4% and 42.8%, respectively, but there was no significant difference in the removal efficiency of heavy metals per gram of dry weight bacteria, attributed to the enhanced survival of *E. coli*/pET-28a-*TtCSA1*.

### 2.6. Removal of Cadmium Ions from Simulated Wastewater

**Figure 8:**
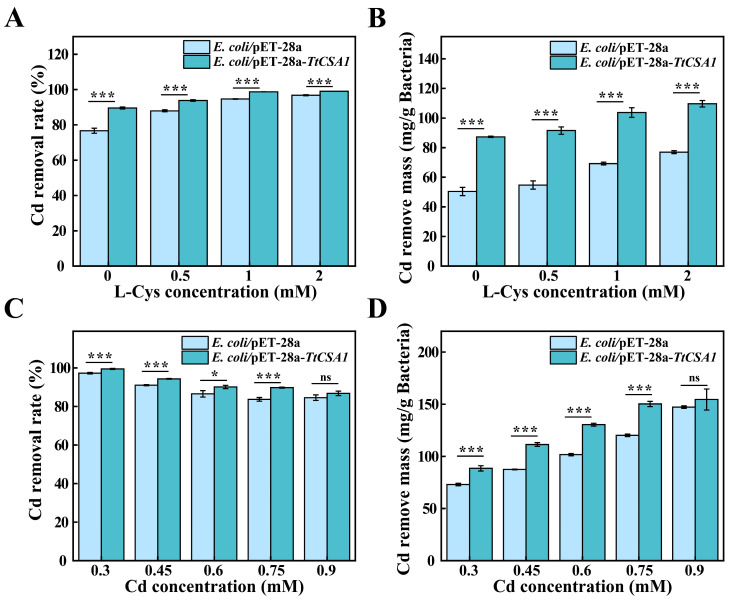
*E. coli*/pET-28a-*TtCSA1* effectively removed cadmium in wastewater. (**A**) Removal rates of cadmium by *E. coli* strains in wastewater containing 0.3 mM Cd^2+^, with the addition of 0, 0.5, 1, and 2 mM L-cysteine. (**B**) Mass of cadmium removed per gram of dry weight of the biomass from the experiments in (**A**). (**C**) Removal rates of cadmium by *E. coli* strains in wastewater containing 2 mM L-cysteine, with varying concentrations of Cd^2+^ (0.3, 0.45, 0.6, 0.75, and 0.9 mM). (**D**) Mass of cadmium removed per gram of dry weight of the biomass from the experiments in Figure C. The symbols ns, *, and *** represent non-significance (*p* > 0.05), significant differences (*p* < 0.05), and extremely significant differences (*p* < 0.01), respectively.

The removal rates of cadmium in simulated wastewater were further assessed under different conditions. The removal rates of *E. coli*/pET-28a-*TtCSA1* and *E. coli*/pET-28a for 0.3 mM cadmium were 89.5% and 76.6%, respectively (Figure 8A). *E. coli*/pET-28a-*TtCSA1* also had a higher removal mass of Cd^2+^ per gram of dry weight bacteria than *E. coli*/pET-28a (Figure 8B) and exhibited higher proliferation capacity compared to *E. coli*/pET-28a (Figure S6). With the addition of 2 mM L-cysteine, the removal rates of *E. coli*/pET-28a-*TtCSA1* and *E. coli*/pET-28a reached 99.0% and 96.8%, respectively. Subsequently, the removal rates of different concentrations of cadmium ions were assessed under 2 mM L-cysteine addition. The removal rates of 0.3, 0.45, 0.6, and 0.75 mM Cd^2+^ by *E. coli*/pET-28a were 97.2%, 91.0%, 86.5% and 83.7%, respectively. The removal mass of Cd^2+^ per gram of dry weight bacteria was 73.0 mg, 87.5 mg, 100.6 mg, and 121.7 mg, respectively. The removal rates of 0.3, 0.45, 0.6, and 0.75 mM Cd^2+^ by *E. coli*/pET-28a-*TtCSA1* were 99.4%, 94.3%, 90.1%, and 89.8%, respectively. The removal mass of cadmium ions per gram dry weight was 88.6 mg, 111.4 mg, 130.4 mg and 150.3 mg, respectively. The removal rates of cadmium by *E. coli*/pET-28a-*TtCSA1* were higher than those in *E. coli*/pET-28a (Figure 8C,D).

## 3. Discussion

The pathways associated with H_2_S synthesis play a crucial role in cellular responses to cadmium stress [40,41]. In *Arabidopsis thaliana*, overexpression of the hydrogen sulfide-related gene *AtLCD* enhances the organism’s ability to cope with cadmium stress [42]. *E. coli* overexpressing cysteine desulfurase produces increased amounts of sulfide, which facilitates cadmium precipitation [43]. Overexpression of the glutathione gene *LmGSTF3* in *Lemna minor* significantly enhances its capacity to withstand cadmium stress [44]. Our previous studies demonstrated that reduced expression of *TtCSA1* in *T. thermophila* significantly impacts cellular responses to cadmium stress [36]. In this study, we found that overexpression of TtCsa1 in *E. coli* significantly improved cellular survival in rich medium under cadmium stress (Figure 1). Furthermore, *E. coli*/pET-28a-*TtCSA1* overexpressing TtCsa1 exhibited greater cadmium tolerance in wastewater conditions (Figure S6). 

Biomineralization leading to the formation of CdS is a crucial mechanism that enables microorganisms to cope with cadmium stress [16,35]. TtCsa1 has been identified as an enzyme that utilizes cysteine to produce H_2_S [34]. In the presence of cysteine, cells primarily alleviate cadmium toxicity through biomineralization, resulting in the generation of CdS [26]. In this study, the addition of L-cysteine promotes CdS QD formation in *E. coli*/pET-28a-*TtCSA1*, with stronger fluorescence intensity and a faster production rate (Figure 2). This enhancement is attributed to the rapid generation of H_2_S (Figure 4). The CdS QDs were also produced in the extracellular aquatic environment and exhibited a red shift with prolonged incubation time. This phenomenon indicates an increase in the particle size of CdS QDs (Figure 3) [45]. This change parallels CdS QDs generated through a single enzyme system in vitro [36]. These findings suggest that the CdS QDs undergo aggregation, leading to an increase in particle size. Transmission electron microscopy (TEM) analyses further confirmed the presence of CdS QDs and aggregates around the cell surface (Figure 5). The CdS produced in LB showed a granular distribution, while the CdS produced in wastewater showed a large mass distribution, which may be caused by the high rate of cell absorption of cadmium and cysteine in wastewater.

Under rich medium conditions, *E. coli*/pET-28a-*TtCSA1* demonstrated superior removal efficiency across different cadmium concentrations ranging from 0.3 to 0.6 mM (Figure 7). This enhanced efficiency is attributed to the expression of TtCsa1.TtCsa1 rapidly catalyzes cysteine to generate H_2_S, which serves as a critical substrate for biomineralization and subsequent CdS formation. The biosynthesis of CdS QDs facilitates cadmium bioremoval. Importantly, *E. coli*/pET-28a-*TtCSA1* maintained a higher cadmium removal rate in simulated wastewater (Figure 8). Under nutrient conditions, the cadmium removal rate by *Bacteria* G303 was 94.7% for 0.4 mM cadmium within 96 h [17]. The *E. coli*/pLC67 strain achieved a removal rate of 99% for 0.4 mM cadmium within 48 h [43]. Additionally, the deep-sea bacterium *Pseudomonas stutzeri* 273 demonstrated a 76% removal rate for 0.4 mM cadmium within 24 h [16]. In wastewater, the alga *Dunaliella salina* showed an 11.3% removal rate for 0.66 mM cadmium over 24 h [46]. These results indicate that *E. coli*/pET-28a-*TtCSA1* possesses comparable cadmium removal capabilities to those reported in existing bioremediation technologies. Compared to other biological methods, the *E. coli*/pET-28a-*TtCSA1* exhibited a stronger cadmium removal rate in wastewater than under nutrient-rich conditions. This enhanced removal efficiency may be attributed to the robust cadmium resistance of *E. coli*/pET-28a-*TtCSA1* in wastewater, whereas other organisms might experience a decline in cadmium tolerance due to nutrient deficiency in such environments. In the presence of cysteine, the increased production of H_2_S by *E. coli*/pET-28a-*TtCSA1* directly facilitates the conversion of cadmium into less toxic CdS. This process significantly reduces the intracellular concentration of free cadmium ions. Given its efficiency and the ease of genetic modification, *E. coli*/pET-28a-*TtCSA1* presents significant potential for applications in the removal of the environmental heavy metal cadmium. (Table S1).

Cadmium-contaminated water environments often contain other pollutants such as sulfates and phosphates [47,48]. Sulfate-reducing bacteria can effectively remove sulfate pollution from wastewater while generating S^2^⁻, which can subsequently react with cadmium to form cadmium sulfide [49]. This process is facilitated by the presence of sulfate reductases in these bacteria, including adenosine-5’-phosphosulfate reductase and sulfite reductase [50,51,52]. However, sulfate-reducing bacteria are strict anaerobes, which necessitate the creation of anaerobic environments for their application [53]. To enhance the application potential of sulfate-reducing bacteria in aerobic environments, expressing sulfate-reducing enzymes in *E. coli* may be a viable approach. Such genetically modified bacteria, through the overexpression of sulfate reductases, would possess enhanced capabilities for sulfate removal while simultaneously removing cadmium contamination. Given the diverse extreme environments in nature, the presence of various enzymes that confer resistance to these conditions suggests that expressing enzymes from other organisms in engineered strains may offer promising applications.

In summary, the heterologous expression of TtCsa1 in *E*. *coli* enhances its tolerance to cadmium and increases H_2_S production with the addition of cysteine. The resulting H_2_S subsequently participates in a biomineralization reaction with cadmium, leading to the formation of CdS QDs. This approach facilitates the effective bioremediation of cadmium from aquatic environments. The engineered *E. coli*/pET-28a-*TtCSA1* possesses a greater advantage for cadmium bioremediation in both rich medium and wastewater.

## 4. Materials and Methods

### 4.1. Strains, Media, and Reagents

The DNA sequence of *TtCSA1* was codon-optimized and cloned into the pET-28a plasmid, resulting in the construction of the plasmid pET-28a-*TtCSA1* [34]. The plasmids pET-28a-*TtCSA1* and pET-28a were transformed into *Escherichia coli* BL21 (DE3), and positive clones were screened to obtain the *E. coli*/pET-28a-*TtCSA1* and *E. coli*/pET-28a strains [34]. The strains were cultured in LB medium (rich medium, 10 g/L peptone, 10 g/L NaCl, 5 g/L yeast extract, pH 7.4) at 37 °C with shaking at 180 rpm (Orbital shaker TS-2, Haimen Kylin-Bell Lab Instruments Co. Ltd,Jiangsu, China). Simulated wastewater was prepared using water collected from the Fen River in Taiyuan, which was added with cadmium and equilibrated overnight. L-cysteine (Sangon Biotech Co., Ltd., Shanghai, China) and cadmium chloride (Beijing Solaibao Technology Co., Ltd., Beijing, China) were prepared as stock solutions using ultrapure water.

### 4.2. Determination of Growth Curves and Minimum Inhibitory Concentration of Cadmium Ions

*E. coli*/pET-28a and *E. coli*/pET-28a-*TtCSA1* were cultured overnight and inoculated into LB medium at a 1:100 volume ratio. The cultures were incubated at 37 °C until the optical density at 600 nm (OD_600_) reached 0.6. Adding 0.05mM IPTG induced protein expression for 24 h. Subsequently, the cells were inoculated into 10 mL of LB medium, maintaining a cell concentration of OD_600_ at 0.05, and the OD_600_ was measured at regular time intervals to monitor bacterial growth. To determine the minimum inhibitory concentration (MIC) of cadmium ions on the strains, *E. coli*/pET-28a and *E. coli*/pET-28a-*TtCSA1* were cultured to an OD_600_ of 0.6, followed by the addition of 0.05 mM IPTG to induce protein expression at 16 °C for 24 h. After induction, the cells were inoculated into 10 mL of LB medium with an initial OD_600_ 0.05, and cadmium chloride was added at concentrations of 0,0.15, 0.3, 0.45, 0.6, 0.75, and 0.9 mM. After 24 h of incubation at 37 °C, the OD_600_ of the cultures was measured.

To detect the growth curve of cells in simulated wastewater, water samples were collected from the Fen River in Taiyuan and added with cadmium to simulate wastewater [54]. Cells with induced protein expression were suspended in the wastewater, maintaining a cell concentration of OD_600_ at 1.1–1.2. After adding cadmium, the optical density of bacteria was measured at regular intervals.

### 4.3. Preparation of E. coli/CdS and Fluorescence Intensity Analysis

After protein induction, *E. coli*/pET-28a and *E. coli*/pET-28a-*TtCSA1* were collected using centrifugation at 12,300 g for 10 min (Centrifuge 5804R, Eppendorf, Hamburg, Germany). The cell pellets were resuspended in 10 mL of fresh medium, maintaining a cell density of OD_600_ at 1.1–1.2. L-cysteine and cadmium ions were then added to the suspension to promote the intracellular synthesis of CdS QDs. The cultures were incubated at 37 °C with shaking at 180 rpm, and the formation of CdS QDs within the cells was monitored at regular time intervals.

To analyze the fluorescence properties of *E. coli*/CdS, the cells were collected by centrifugation at 6150 g for 1 min, washed twice with PBS, and resuspended. Fluorescence microscopy (BX51, OLYMPUS, Tokyo, Japan) was employed to observe the cells under UV light excitation, focusing on the fluorescence characteristics of CdS QDs within the cells. The fluorescence intensity of the cells was quantified using ImageJ software (V1.80.), providing a detailed analysis of the fluorescence characteristics [40].

### 4.4. Production and Detection of H_2_S

To investigate the production of H_2_S, *E. coli*/pET-28a and *E. coli*/pET-28a-*TtCSA1* were collected after IPTG induction, resuspended, and adjusted to an OD_600_ of 1.1–1.2. A 5 mL aliquot of the cell suspension was transferred to a 15 mL test tube. To examine the effect of L-cysteine concentration on H_2_S production, cadmium ions and varying concentrations of L-cysteine (0, 0.2, 0.5, 1, 2, and 4 mM) were added to the system. Similarly, to evaluate the impact of cadmium ion concentration on H_2_S production, L-cysteine and different concentrations of cadmium ions (0, 0.15, 0.3, 0.45, 0.6, 0.75, and 0.9 mM) were added. A pre-moistened lead acetate test strip was placed 4 cm above the top of the test tube, and the tube cap was tightly sealed. The test tubes were incubated at 37 °C in a shaking incubator, and the lead acetate test strip was periodically observed for color changes [55].

### 4.5. The Absorption and Emission Spectrum of CdS QDs

*E. coli*/pET-28a and *E. coli*/pET-28a-*TtCSA1* were centrifuged at 12,300 g for 10 min, and the supernatant was collected for analysis. The collected supernatant was used to analyze the ultraviolet absorption spectrum of cadmium sulfide QDs in the reaction system using a UV-visible spectrophotometer. Fluorescence emission spectra at an excitation wavelength of 350 nm were measured using a fluorescence spectrophotometer [56].

### 4.6. Characterization of CdS QDs by Transmission Electron Microscope and Energy Dispersive X-Ray Spectroscopy (EDX)

After protein induction, *E. coli*/pET-28a and *E. coli*/pET-28a-*TtCSA1* were centrifuged at 12,300 g for 10 min, and the cells were resuspended in an equal volume of fresh medium to an OD_600_ of 1.1–1.2. The suspension was incubated with 0.3 mM cadmium chloride and 0.5 mM L-cysteine for 48 h, and the cells were collected at 12,300 g for 2 min. The cells fixed with 2.5% glutaraldehyde were loaded onto carbon film-coated copper grids by immersion, washed once with ultrapure water, and dried using blotting paper. The cells were then observed by a transmission electron microscope (JEOL JEM-F200, Hitachi, Tokyo, Japan). Energy dispersive X-ray spectroscopy (EDX) analysis was conducted at an accelerating voltage of 15 kV for 100 s [57]. For Scanning Electron Microscope (SEM) observation, the cells were washed twice with PBS and fixed with 2.5% glutaraldehyde at 4°C for 20 min. The cell pellets were washed with 100 mM PBS. Dehydration was performed using a graded ethanol series (30%, 50%, 70%, 90%, 95%, and absolute ethanol) for 10 min each. The cell suspension was placed on a lysine-coated coverslip, mounted on stubs, and sputter-coated with gold and platinum (10 nm) using a KAS-2000F ion sputter coater for 5 min. Then, the samples were observed using SEM (ZEISS Sigma 300, Jena, Germany).

### 4.7. X-Ray Diffraction of E. coli/pET-28a-TtCSA1-CdS

*E. coli*/pET-28a-CdS and *E. coli*/pET-28a-*TtCSA1*-CdS were washed twice with PBS, and resuspended in a small amount of ultrapure water. The cells were freeze-dried using a lyophilizer and subsequently ground into a powder. X-ray diffraction (XRD) patterns were recorded using an X-ray diffractometer (Panalytical Empyrean, Almelo, Netherlands) [15].

### 4.8. Cadmium Removal in LB Medium and Simulated Wastewater

*E. coli*/pET-28a-*TtCSA1* and *E. coli*/pET-28a were collected after induction and washed twice with PBS. The cells were then resuspended in 10 mL of fresh LB medium in a 100 mL Erlenmeyer flask, maintaining an OD_600_ of 1.1–1.2. The cultures were supplemented with 0.5 mM L-cysteine and varying concentrations of cadmium ions (0.15, 0.3, 0.45, 0.6, and 0.75 mM) and incubated for 48 h. The cells were centrifuged at 12,300 g for 10 min. The supernatant was collected, and the cadmium content in the medium was determined using Inductively Coupled Plasma Optical Emission Spectrometry/Mass Spectrometry (ICP-OES/MS, Agilent 5110 (OES), CA,USA). The cell pellets were dried at 37 °C for 48 h to determine the biomass dry weight. The cadmium removal efficiency and adsorption capacity were calculated using the following equations:

*R* = (1 − *C*/*C_0_*) × 100% (1)

*E_e_* = *q*/*m* (2)

where *R* is the bacterial cadmium removal efficiency (%), *C_0_* is the initial cadmium concentration (mg/L), and *C* is the cadmium concentration in the culture supernatant after 48 h of bacterial growth (mg/L). *E_e_* is the cadmium adsorption capacity of the bacteria (mg/g), *q* is the mass of cadmium adsorbed by the bacteria (mg), and *m* is the biomass weight (g) [7].

To investigate cadmium removal from wastewater. The cells were washed twice with PBS and resuspended to maintain an OD_600_ of 1.1–1.2. The cells were recollected by centrifugation and resuspended in simulated wastewater containing 0.3 mM cadmium ions in a 100 mL Erlenmeyer flask. Different concentrations of L-cysteine (0, 0.5, 1, 2, and 4 mM) were added, and the cultures were incubated in a shaker at 37 °C for 48 h. The methods for calculating cadmium removal efficiency and adsorption capacity were identical to those described previously. Additionally, the cells were suspended in simulated wastewater containing cadmium ions (0.3, 0.45, 0.6, 0.75, and 0.9 mM) and 2 mM L-cysteine, incubated in 100 mL Erlenmeyer flasks for 12 h, and cadmium removal efficiency and adsorption capacity were calculated as previously described.

### 4.9. Statistical Analysis

All experiments were conducted in triplicate, and the data are expressed as the mean ± standard deviation of the three experiments. Statistical analysis was performed using one-way analysis of variance (ANOVA). The Tukey’s multiple comparison test was utilized to evaluate significant differences between treatments. Statistical significance is denoted by a single asterisk for *p* < 0.05 and a triple asterisk for *p* < 0.01.

## 5. Conclusions

Heterologous expression of TtCsa1 enhances *Escherichia coli’s* tolerance for cadmium stress. *E. coli*/pET-28a-*TtCSA1* produces higher levels of H_2_S under L-cysteine incubation, resulting in the formation of more CdS QDs. *E. coli*/pET-28a-*TtCSA1* exhibits superior cadmium removal efficiency. It not only improves cadmium tolerance but also enhances cadmium biomineralization from both rich mediums and simulated wastewater. The engineered *E. coli*/pET-28a-*TtCSA1* holds potential for application in cadmium bioremediation in wastewater.

## Data Availability

The additional data supporting the manuscript are available from the corresponding author upon request.

## Supplementary Materials

The supporting information can be downloaded at: https://www.mdpi.com/article/10.3390/ijms26083685/s1. References [7,16,17,24,26,41,43,49,58,59] are cited in the supplementary materials.
