# Supplementary material for: Overexpression of Tetrahymena Cysteine Synthetase 1 Promotes Cadmium Removal by Biosynthesizing Cadmium Sulfide Quantum Dots in Escherichia coli
Source: Int J Mol Sci. 2025 Apr 13;26(8):3685. doi: 10.3390/ijms26083685 (PMC12028156; doi:10.3390/ijms26083685)
Supplement: Supplementary file 1 [file ijms-26-03685-s001.zip › ijms-3545602-supplementary.pdf]

Overexpression of *Tetrahymen*a Cysteine Synthetase 1 Promotes Cadmium Removal by Biosynthesizing Cadmium Sulfide Quantum Dots in *Escherichia coli*

Wenliang Lei [1], Juan Liu [1], Yiwei Liu [1], Jing Xu [1,2,*] and Wei Wang [1,3,*]

1 Key Laboratory of Chemical Biology and Molecular Engineering of Ministry of Education, Institute of Biotechnology, Shanxi University, Taiyuan 030006, China;

2 School of Life Science, Shanxi University, Taiyuan 030006, China

3 Shanxi Key Laboratory of Biotechnology, Taiyuan 030006, China

* Correspondence: xujing@sxu.edu.cn (J.X.), gene@sxu.edu.cn (W.W.)

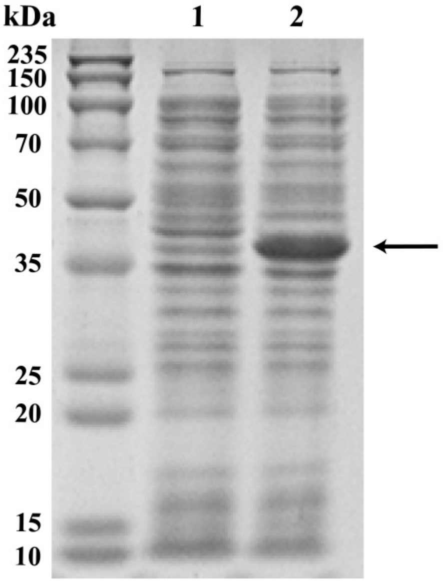

Figure S1. The expression analysis of TtCsa1 in *E. coli*/pET-28a-*TtCSA1*

SDS-PAGE analysis of protein expression in *E. coli*/pET-28a-*TtCSA1*. Cellular lysates of *E. coli*/pET-28a (Lane 1) and *E. coli*/pET-28a-*TtCSA1* (Lane 2) were analyzed. M, Molecular marker. The arrow indicates the position of TtCsa1.

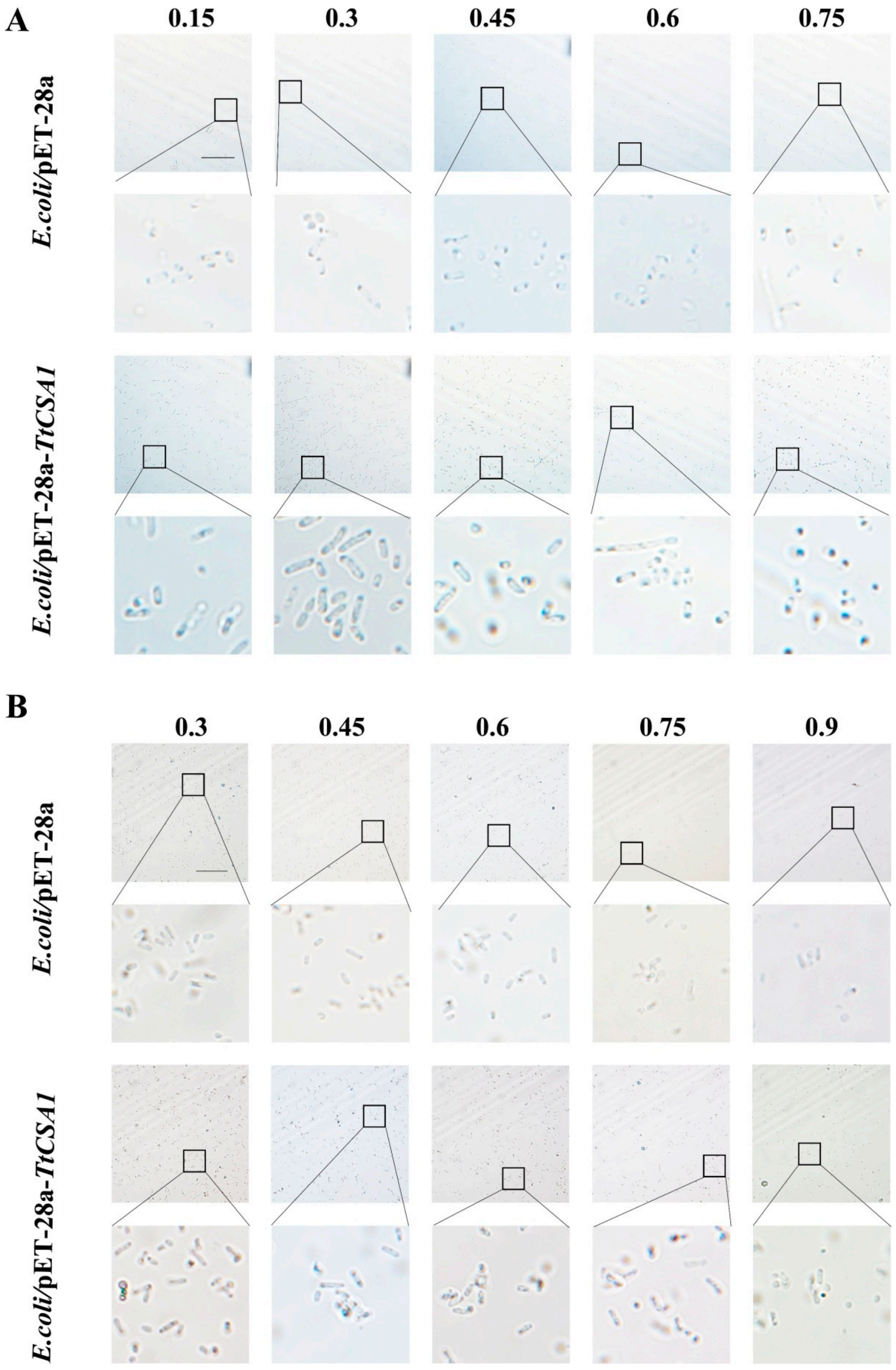

Figure S2. The biomineralization of cadmium sulfide in *E. coli*

Biomineralization of cadmium sulfide by Bright Field Microscopy. (**A**) *E. coli*/pET-28a and *E. coli*/pET-28a-*TtCSA1* were incubated for 48 hours in LB medium containing 0.5 mM L-cysteine and varying cadmium concentrations (0.15, 0.3, 0.45, 0.6, and 0.75 mM). (**B**) *E. coli*/pET-28a and

E. coli/pET-28a-*TtCSA1* were incubated for 12 hours in wastewater containing 1 mM L-cysteine and varying cadmium concentrations (0.3, 0.45, 0.6, 0.75, and 0.9 mM). Scale bar, 20 μm.

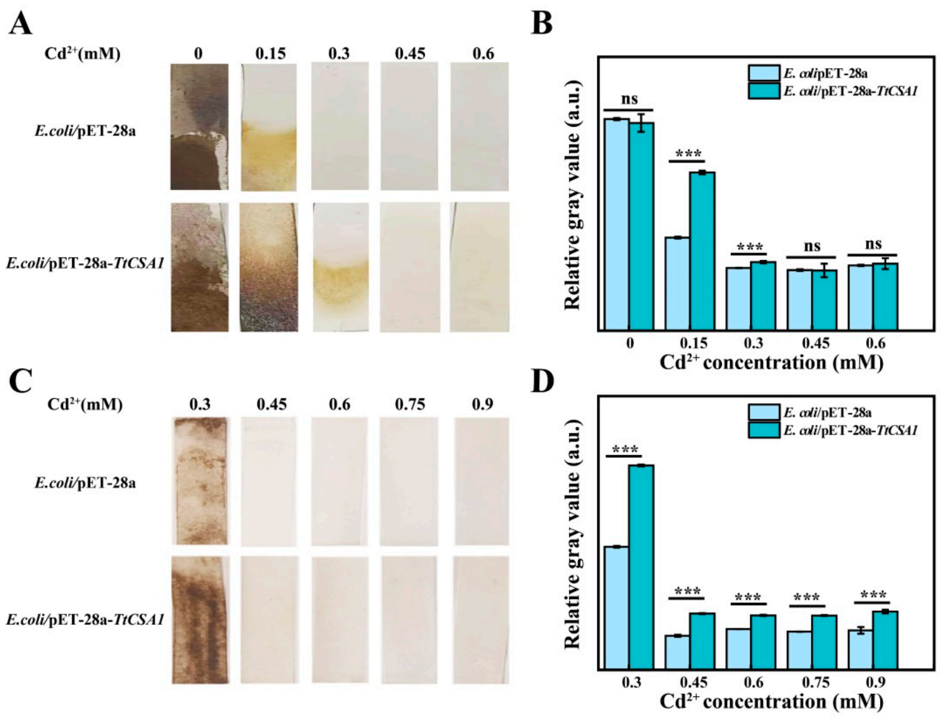

Figure S3. Influence of cysteine concentration on H_2S production in *E. coli*

Detection and analysis of H_2S generation using lead acetate paper. (**A**) Detection of H_2S generation using lead acetate paper after incubating *E. coli*/pET-28a and *E. coli*/pET-28a-*TtCSA1* in LB medium with different concentrations of cadmium ions (0, 0.15, 0.3, 0.45, and 0.6 mM) and 0.5 mM L-cysteine for 12 hours.(**B**) Gray-scale analysis of discoloration of lead acetate test paper in Figure A.(**C**) Detection of H_2S generation using lead acetate paper after incubating *E. coli*/pET-28a and *E. coli*/pET-28a-*TtCSA1* in wastewater with varying concentrations of cadmium ions (0.3, 0.45, 0.6, 0.75, and 0.9 mM) and 1 mM L-cysteine for 24 hours.(**D**) Gray-scale analysis of discoloration of lead acetate test paper in Figure C. ns,* and *** represent non-significance (P > 0.05),significant differences (P < 0.05) and extremely significant differences (P < 0.01).

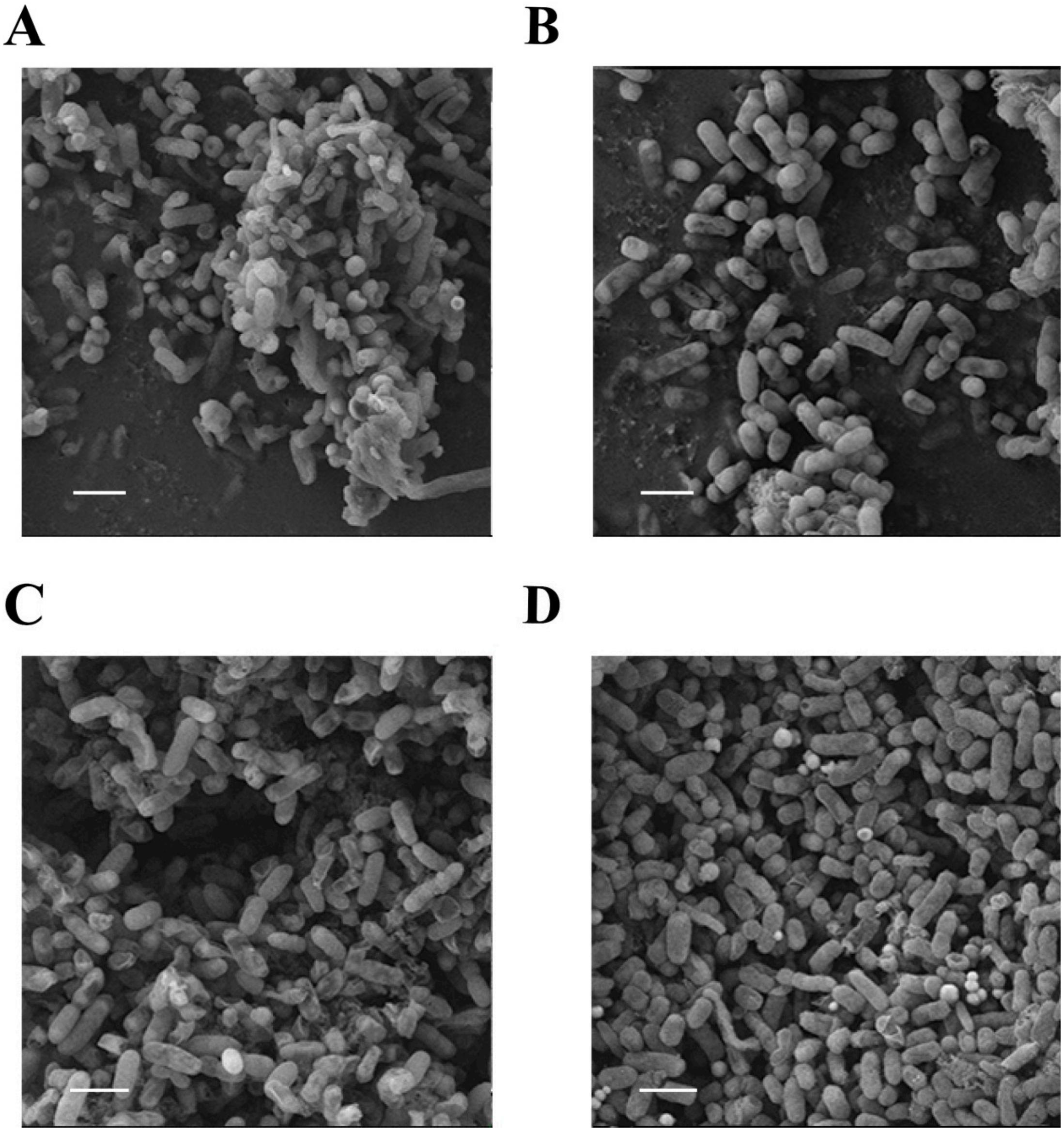

Figure S4. Scanning electron microscope observation of *E. coli* treated with cadmium and cysteine

(**A, B**) The morphology of *E. coli*/pET-28a and *E. coli*/pET-28a-*TtCSA1* was observed by Scanning Electron Microscopy (SEM) after incubation for 48 hours in LB medium containing 0.45 mM cadmium ions and 0.5 mM L-cysteine. Scale bar, 2 μm. (**C, D**) The morphology of *E. coli*/pET-28a and *E. coli*/pET-28a-*TtCSA1* was observed by Scanning Electron Microscopy (SEM) after incubation for 12 hours in wastewater containing 0.6 mM cadmium ions and 1 mM L-cysteine. Scale bar, 2 μm.

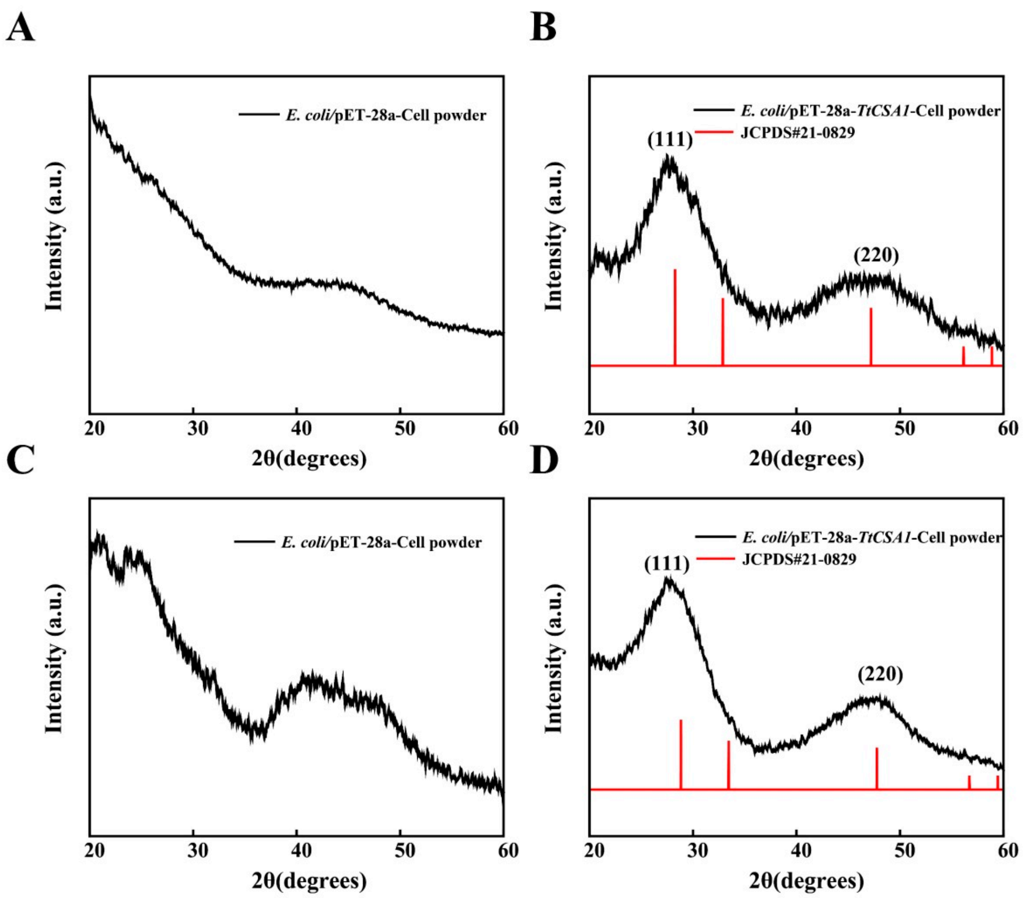

Figure S5. X-ray diffraction analysis of *E. coli*-CdS

(**A, B**) X-ray diffraction (XRD) patterns of *E. coli*/pET-28a-CdS and *E. coli*/pET-28a-*TtCSA1*-CdS. The strains were incubated in LB medium containing 0.45 mM Cd^{2+} and 0.5 mM L-cysteine for 48 hours. (**C, D**) XRD patterns of *E. coli*/pET-28a-CdS and *E. coli*/pET-28a-*TtCSA1*-CdS. The strains were incubated in simulated wastewater containing 0.6 mM Cd^{2+} and 1 mM L-cysteine for 48 hours.

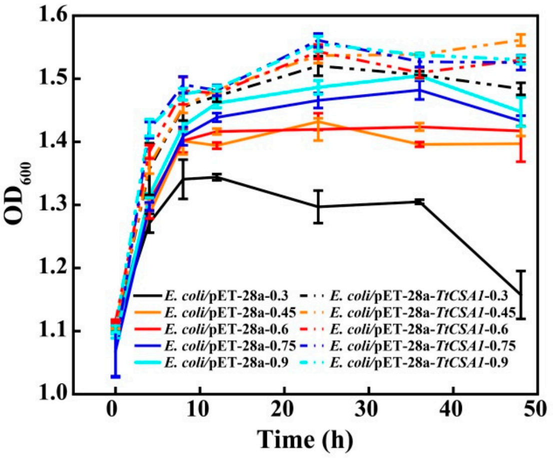

Figure S6. The formation of CdS QD promotes proliferation of *E. coli* in wastewater

The amount of *E. coli*/pET-28a and *E. coli*/pET-28a-*TtCSA1* increased in wastewater containing 0.3, 0.45, 0.6, 0.75, and 0.9 mM Cd^{2+}.

Table S1. Comparative Analysis of the Cadmium Removal Capacity

strain	Initial Cd^{2+} concentration (mM)	Time (h)	Removal rate	removal method	water environment	Reference
Bacteria G303	0.4	96	94.7%	biominerali zation	LB Medium	[1]
Tetrahymena thermophila SB210	0.44	24	80%	biominerali zation	Cd^{2+} solution	[2]
deep-sea bacterium *Idiomarina sp.* OT37-5b	0.4	24	99%	biominerali zation	2216E broth	[3]
Rhodopseudom onas palustris	0.3	24	99%	biominerali zation	medium	[4]
Pseudomonas stutzeri 273	0.4	24	76%	biominerali zation	LB Medium	[5]
E. coli/ pLC67	0.4	48	99%	biominerali zation	LB Medium	[6]
sulfatereducing bacteria	0.7	24	70%	biominerali zation	wastewater	[7]
Aspergillus niger	0.0032	72	79%	bioleaching	Medium	[8]
Pseudomonas putida/ X4	0.11	2	90 %	Biosorption	LB Medium	[9]
Dunaliella salina	0.66	24	11.3%	biosorption	seawater	[10]
E. coli/pET-28a-*TtCSA1*	0.3	48	98.2%	biominerali zation	LB Medium	This Work
E. coli/pET-28a-*TtCSA1*	0.45	12	94.3%	biominerali zation	wastewater	This Work

References

1. Zhang, S.; Song, M.; Zhang, J.; Wang, H. Cysteine and thiosulfate promoted cadmium immobilization in strain G303 by the formation of extracellular CdS. *Sci. Total Environ.* **2024,** *923*, 171457.
2. Tu, J. W.; Li, T.; Gao, Z. H.; Xiong, J.; Miao, W. Construction of CdS-*Tetrahymena thermophila* hybrid system by efficient cadmium adsorption for dye removal under light irradiation. *J. Hazard. Mater.* **2022,** *439*, 129683.
3. Ma, N.; Sha, Z.; Sun, C. Formation of cadmium sulfide nanoparticles mediates cadmium resistance and light utilization of the deep-sea bacterium *Idiomarina* sp. OT37-5b. *Appl. Environ. Microbiol.* **2021,** *23*, 2, 934-948.
4. Xing, S. F.; Tian, H. F.; Yan, Z.; Song, C.; Wang, S. G. Stability and biomineralization of cadmium sulfide nanoparticles biosynthesized by the bacterium *Rhodopseudomonas palustris* under light. *J. Hazard. Mater. Adv.* **2023,** *458*, 131937.
5. Ma, N.; Cai, R.; Sun, C. Threonine dehydratase enhances bacterial cadmium resistance via driving cysteine desulfuration and biomineralization of cadmium sulfide nanocrystals. *J. Hazard. Mater. Adv.* **2021,** *417*, 126102.
6. Wang, C. L.; Lum, A. M.; Ozuna, S. C.; Clark, D. S.; Keasling, J. D. Aerobic sulfide production and cadmium precipitation by *Escherichia coli* expressing the *Treponema denticola* cysteine desulfhydrase gene. *Appl. Microbiol. Biotechnol.* **2001,** *56*, 3-4, 425-30.
7. Ren, W.; Wan, C.; Li, Z.; Liu, X.; Zhang, R.; Yang, X.; Lee, D. J. Functional CdS nanocomposites recovered from biomineralization treatment ofsulfate wastewater and its applications in the perspective of photocatalysis and electrochemistry. *Sci. Total Environ.* **2020,** *742*, 140646.
8. Khan, I.; Aftab, M.; Shakir, S.; Ali, M.; Qayyum, S.; Rehman, M. U.; Haleem, K. S.; Touseef, I. Mycoremediation of heavy metal (Cd and Cr)–polluted soil through indigenous metallotolerant fungal isolates. *Environ. Monit. Assess.* **2019,** *191*, 9.
9. He, X.; Chen, W.; Huang, Q. Surface display of monkey metallothionein α tandem repeats and EGFP fusion protein on Pseudomonas putida X4 for biosorption and detection of cadmium. *Appl. Microbiol. Biotechnol.* **2011,** *95*, 6, 1605-1613.
10. Folgar, S.; Torres, E.; Pérez-Rama, M.; Cid, A.; Herrero, C.; Abalde, J. Dunaliella salina as marine microalga highly tolerant to but a poor remover of cadmium. *J. Hazard. Mater.* **2009,** *165*, 1-3, 486-493.
